# Left ventricular outflow obstruction predicts increase in systolic pressure gradients and blood residence time after transcatheter mitral valve replacement

**DOI:** 10.1038/s41598-018-33836-7

**Published:** 2018-10-19

**Authors:** Adelaide De Vecchi, David Marlevi, David A. Nordsletten, Ioannis Ntalas, Jonathon Leipsic, Vinayak Bapat, Ronak Rajani, Steven A. Niederer

**Affiliations:** 1 Department of Biomedical Engineering, School of Imaging Sciences & Biomedical Engineering, King’s College London, King’s Health Partners, St Thomas Hospital, London, SE1 7EH UK; 2 School of Technology and Health, KTH Royal Institute of Technology, Hälsovägen 11C, 141 52 Huddinge, Sweden; 3 Department of Clinical Sciences, Danderyd Hospital, Karolinska Institutet, 17177 Stockholm, Sweden; 4 Department of Cardiology, Guy’s and St Thomas’ NHS Foundation Trust, London, UK; 5 Department of Radiology and Medicine, University of British Columbia, Vancouver, British Columbia Canada; 6 Department of Surgery, Columbia University Medical Center, New York, NY 10032 USA

## Abstract

Left ventricular outflow tract (LVOT) obstruction is a relatively common consequence of transcatheter mitral valve replacement (TMVR). Although LVOT obstruction is associated with heart failure and adverse remodelling, its effects upon left ventricular hemodynamics remain poorly characterised. This study uses validated computational models to identify the LVOT obstruction degree that causes significant changes in ventricular hemodynamics after TMVR. Seven TMVR patients underwent personalised flow simulations based on pre-procedural imaging data. Different virtual valve configurations were simulated in each case, for a total of 32 simulations, and the resulting obstruction degree was correlated with pressure gradients and flow residence times. These simulations identified a threshold LVOT obstruction degree of 35%, beyond which significant deterioration of systolic function was observed. The mean increase from baseline (pre-TMVR) in the peak systolic pressure gradient rose from 5.7% to 30.1% above this threshold value. The average blood volume staying inside the ventricle for more than two cycles also increased from 4.4% to 57.5% for obstruction degrees above 35%, while the flow entering and leaving the ventricle within one cycle decreased by 13.9%. These results demonstrate the unique ability of modelling to predict the hemodynamic consequences of TMVR and to assist in the clinical decision-making process.

## Introduction

With the success of transcatheter aortic valve (AV) implantation for patients at high surgical risk, attention has now been cast on transcatheter therapies for the mitral valve (MV)^1^. These techniques vary from balloon and self-expanding AV used in the mitral position, to newer devices specific to the MV, to Valve-in-Valve interventions for failed bioprosthetic devices^2–4^. Irrespective of the approach, it is clear that transcatheter mitral valve replacement (TMVR) poses a distinct set of challenges compared to AV implantation. The geometry of the mitral apparatus and its relationship to atrial and ventricular structures introduce significant concerns pertaining to device stability. Specifically, TMVR devices need to be oversized to ensure adequate anchoring while accommodating deformational changes of the valve annulus during the cardiac cycle. This, in conjunction with the displacement of the anterior MV leaflet towards the AV, introduces a risk of left ventricular outflow tract (LVOT) obstruction as it involves a taller frame protruding inside the left ventricle (LV)^5,6^.

The interplay between the protruding device, the septal wall, and the anterior leaflet results in the formation of a neo-LVOT with reduced cross-sectional area compared to the original outflow tract^7^. Outflow obstruction can in turn increase left ventricular afterload and introduce adverse hypertrophic LV remodeling, which correlates with high risks of ventricular failure especially in patients with underlying dysfunction^8^. Evidence also exists that TMVR is associated with risks of blood stagnation inside the ventricle, with elevated pressure gradients and less effective ejection after MV implantation identified as predisposing factors for early postoperative thrombi^9–11^.

Although pre-procedural planning aims to predict the likelihood of LVOT obstruction, existing techniques are largely based on standard geometric evaluation of pre-procedural imaging datasets and there is consensus on neither the effect of the cardiac phase at which measurements are taken, nor the procedural canting of the device on the prediction. It also remains unknown as to what threshold of LVOT obstruction is clinically significant in these patients. To enable a successful introduction of TMVR devices, it is clear that careful patient selection and advanced quantitative evaluation of both anatomy and hemodynamics will be vital. Advanced imaging evaluation can provide valuable insight into the patient pathophysiology^12–15^. In the current study we sought to evaluate the utility of combining this technique with computer models to simulate blood flow dynamics in TMVR patients and to predict the ventricular response to LVOT obstruction.

## Methods

### Patient data

Seven patients were included in the study (Tables 1 and 2): six were under consideration for TMVR with bioprosthetic AV devices, e.g. Sapien (Edwards Lifesciences, Irvine, CA, USA) and Lotus (Boston Scientific, Marlborough, MA, USA), and one was assessed for treatment under compassionate use with an Intrepid valve (Medtronic, Minneapolis, MN, USA). Patients proceeded to transapical implantation with the chosen device, except Cases 2 and 7 – unimplanted due to heart failure and predicted excessive LVOT obstruction, respectively. This study complies with the principles of the Declaration of Helsinki and the research protocol was approved by local ethics committees (Guy’s and St Thomas’ NHS Foundation Trust); all patients provided informed consent. Datasets were collected pre-operatively only, except Case 6 where the complete acquisition protocol was also applied post-TMVR for model validation.

**Table 1:** Baseline patient characteristics.

Measurement	Pre-TMVR (n = 7)
Male	4 (57)
Age (yrs)	69.5 ± 11.7
Body Mass Index (kg/m^3^)	22.7 ± 2.7
EF (%)	43.4 ± 14.9
LV AR	2.0 ± 0.57
α angle (°)	139.6 ± 15.9
C-sept (mm)	32.2 ± 7.3
MV area (mm^2^)	729 ± 342
MAC	3 (43)
FMR	2 (29)
VIV	1 (14)
VIR	1 (14)

Values are in n (%) or mean ± SD.

EF = ejection fraction; LV AR = left ventricular aspect ratio at end-systole; α = angle of the aorto-mitral junction at end systole; C-sept = distance between the mitral valve coaptation point and the interventricular septum at end-systole; MV = mitral valve; MAC = valve in calcified mitral annulus. FMR = functional mitral regurgitation. VIV = valve in valve. VIR = valve in ring.

**Table 2:** Individual patient characteristics at baseline.

	Height (m)	Weight (Kg)	Body mass index (kg/m^2^)	MV area (mm^2^)	Comorbidities
Case 1	1.70	64.0	22.1	444.2	CKD, COPD, CABG, calcified aortic/mitral annulus
Case 2	1.67	69.0	24.7	1260.0	AF, heart failure (DCM)
Case 3	1.63	73.5	27.7	355.6	MV and TV repair, CABG, PFO repair, PPM
Case 4	1.70	63.0	21.8	858.5	TAVI, IHD with PCI to RCA, PPM for complete heart block, CKD
Case 5	1.60	55.0	21.5	470.0	Tricuspid annuloplasty, minor coronary disease in LAD
Case 6	1.62	56.0	21.3	1060.0	CABG × 3, DM type 2, Hypercholesterolemia, HTN
Case 7	1.60	50.0	19.5	655.0	HTN, CKD Stage 3, AF

CKD = chronic kidney disease. COPD = chronic obstructive pulmonary disease. CABG = coronary artery bypass grafting. NHL = Non-Hodgkin lymphoma. PFO = patent foramen ovale. PPM = permanent pacemaker. AF = atrial fibrillation. DCM = Dilated cardiomyopathy. IHD = ischaemic heart disease. PCI = percutaneous coronary intervention. TAVI = transcatheter aortic valve replacement. RCA = right coronary artery. AVR = aortic valve replacement. MVR = mitral valve replacement. MV = mitral valve. LAD = left ascending artery. DM = diabetes mellitus. HTN = hypertension.

### Imaging protocol

Multiphase contrast-enhanced computed tomography angiograms (CTA) were acquired in all patients using a Philips Brilliance iCT 256-slice MDCT scanner (Philips Healthcare, Best, The Netherlands). Intravenous metoprolol was used to achieve a heart rate of <65 beats/min (or <100 beats/min if in atrial fibrillation). Intravenous contrast (Omnipaque, GE Healthcare, Princeton, NJ, USA) was subsequently injected into the antecubital vein (5 ml/s for a total of 100 ml). Ascending aorta contrast triggered, retrospectively ECG-gated scanning with no dose modulation was performed in a single breath hold after 10–12 seconds with a heart rate-dependent pitch of 0.2–0.45, a gantry rotation time of 270 ms, a tube voltage of 100 or 120 kVp (depending on the patient’s body mass index) and a tube current of 125–300 mA (depending upon the thoracic circumference). Transthoracic and transesophageal echocardiography data were also acquired. The direction and maximum magnitude of blood velocity across the valves was evaluated from Color Doppler data, which was also used to estimate mean and peak LVOT and transaortic pressure gradients. For all Doppler measurements, the average of three signals was taken. The mitral valve annulus was defined as the hinge points between the left atrium and mitral valve leaflets. The area of the annulus was measured as an average of three estimates on a 3D volume acquisition of the left atrium and left ventricle in mid systole by multiplanar reformatted imaging.

### Model personalisation

**Figure 1:**
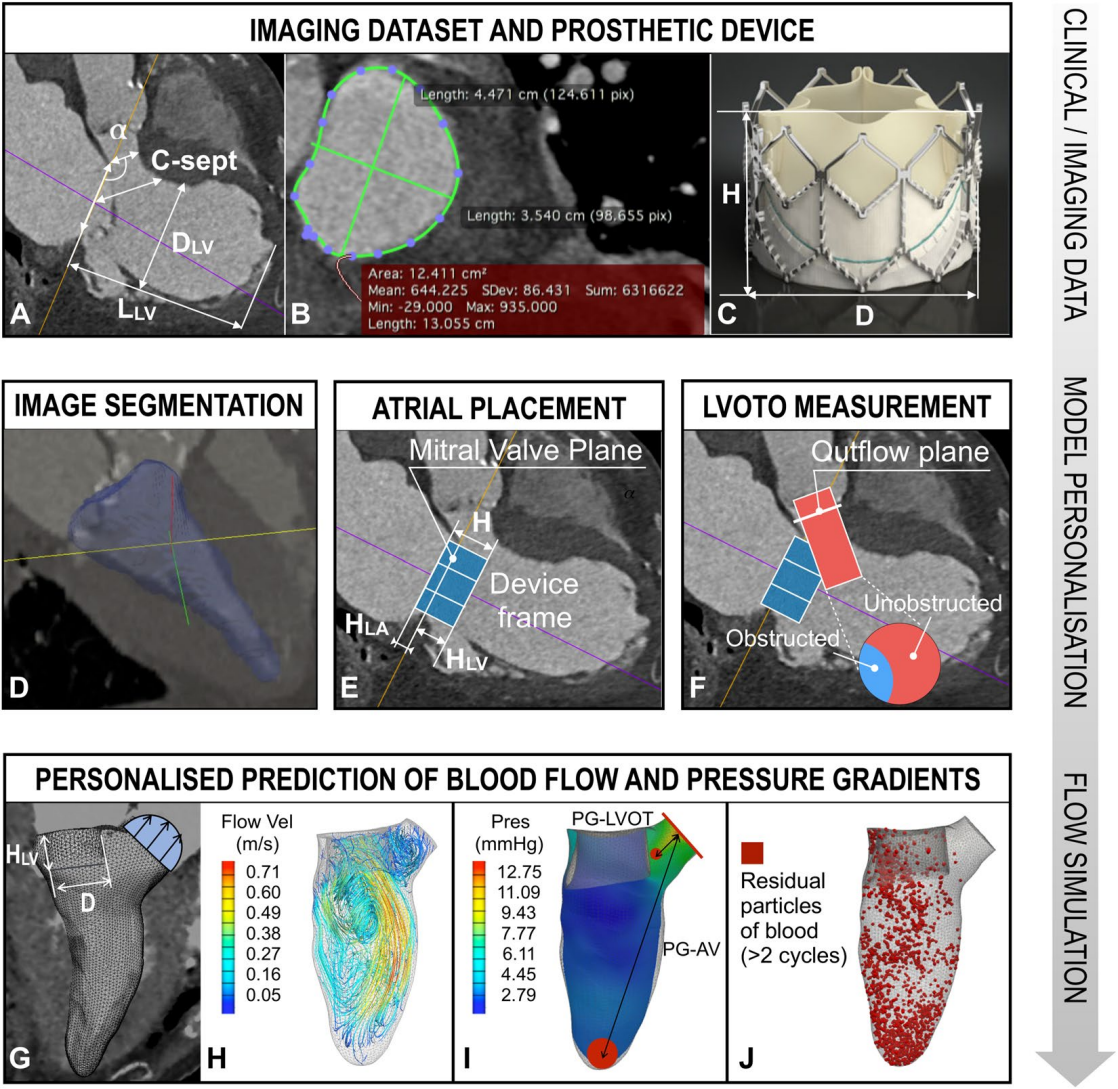
Modeling workflow. (**A**–**C)** Imaging data processing to extract patient anatomy and valve size. (**D**–**F**) Image segmentation, device positioning, and LVOT obstruction calculation. (**G**) Imposition of image-derived boundary conditions and valve parameters for model personalisation. (**H**–**J**) Personalised flow simulations: blood streamlines colored by velocity magnitude (**H**) pressures isocontours (**I**) with measuring sites marked in red; flow component analysis to determine the blood residence time in the LV (**J**).

The valve dimensions, the angle between the MV and AV planes (α), the distance between the MV coaptation point and the interventricular septum (C-sept), and LV aspect ratio (AR = L_LV_/D_LV_) were measured as shown in Fig. 1A–C.

Although the valve dimensions in the models were informed from specific devices, our results can be applied to any stent-based valve design within these size ranges. This study aims at identifying the degree of obstruction that causes significant changes in hemodynamic function, and not at investigating the valve design itself: therefore we chose a simplistic representation of the bioprostheses, with a rigid cylinder of diameter D and height H, respectively equal to the inner diameter and frame height of the commercial valve under consideration for implantation (Fig. 1C). A tetrahedral volume mesh of the LV was created based on the blood pool obtained from the manual segmentation of the LV cavity from CTA data (Fig. 1D). The portion of the valve frame lying inside the LV (H_LV_) is given by:

1 $${H}_{LV}=H-{H}_{LA}$$

where H_LA_ is the portion of the valve frame in the left atrium relative to the mitral sewing cuff (Fig. 1E). H_LA_ was chosen to vary between 3 and 5 mm, resulting in a range of H_LV_ tested for each patient, in agreement with surgical recommendations to prevent late atrial migration^16,17^. The outflow obstruction fraction (OF) was then calculated as the normal projection of the valve frame on the AV plane, divided by the total outflow area (blue and red area respectively in Fig. 1F). In each model, boundary conditions were personalised by extracting information on wall and blood velocity from imaging data (Fig. 1G). Specifically, the wall velocity is derived from the wall motion tracked using an algorithm based on the temporal-sparse free form deformation^18^ and the transvalvular blood velocities were obtained from Doppler ultrasound data at the valve planes^19,20^. The finite-element software CHeart, which has been extensively validated for cardiovascular simulations^21^, was used in all flow simulations to compute the blood pressure and velocity inside the LV (Fig. 1H–J).

### Personalised CFD simulations

Different simulations were performed in each patient: first, the cardiac cycle was simulated in a model without implanted valve to provide baseline conditions, then additional simulations were performed using predictive models where a simplified valve of the same size of the commercial device under consideration was embedded in the LV mesh with varying levels of H_LA_ (Table 3). In each scenario, three consecutive cardiac cycles were simulated to minimise any artifacts from initial conditions. The study comprised a total of 32 personalised simulations for 7 patients. The heterogeneity of this cohort allowed us to test the models under different conditions. In Cases 1 to 5, the anatomical characteristics of the LV allowed H_LV_ to be increased above the frame height H of the commercial device without interfering with the endocardium. These “virtual” scenarios, effectively corresponding to a negative H_LA_ in Eq. 1, were tested to assess the impact of a longer channel on the blood flow dynamics and are marked with an asterisk in Table 3. In Case 7, which was not implanted due to interference of the bioprosthesis with the septal wall, valve heights smaller than the commercially available sizes were tested.

**Table 3:** Modelling characteristics.

	Device size (mm)	Atrial placement H_LA_ (mm)	OF (%)
Case 1	D26/H17	5; 3; 0; −5*	19.0; 40.3; 49.3; 62.6
Case 2	D29/H19	5; 0; −5*	0.0; 0.0; 0.0
Case 3	D26/H17	5; 3; 0; −3*	10.7; 23.2; 29.2; 39.3
Case 4	D27/H17	5; 3; 0; −3*	6.8; 27.4; 38.5; 67.0
Case 5	D27/H17	5; 3; 0; −3*	12.2; 34.6; 43.7; 51.0
Case 6	D27/H17	5; 3; 0	0.0; 0.0; 6.0
Case 7	D23/H10*	5; 3; 1; 0	30.1; 45.0; 51.9; 58.7

D/H = valve diameter/height. OF = obstruction fraction.

*Indicates valve parameters that are different from commercially available sizes and/or routine positioning.

### Anatomical and hemodynamics measurements

**Figure 2:**
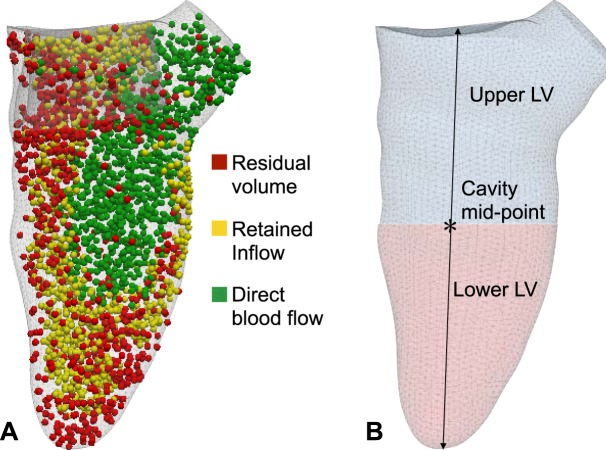
LV flow analysis. (**A**) Subdivision of the LV cavity into upper and lower LV based on the location of the cavity mid-point (dynamically calculated during the cardiac cycle). (**B**) Particle-tracking throughout the cardiac cycle, with flow components labeled according to the residency time in the LV.

The pressure gradients in the LV were calculated at peak systole in each model. In addition to pressure calculations, a functional flow analysis was performed by splitting the blood flow into different components depending on the time spent inside the LV (Fig. 2B), with each component expressed as percentage of the end-diastolic volume^22–24^. The following hemodynamic parameters were tested:

1. *PG-AV (mmHg)*: gradient between the mean pressures within a sphere of radius 4 mm in the apical region and on the AV plane (Fig. 1I).
2. *PG-LVOT (mmHg)*: gradient between the mean pressures within a sphere of radius 1 mm placed 6 mm below the AV plane and on the AV plane (Fig. 1I).
3. *Direct flow (DF, %)*: percentage of blood entering and exiting the LV within the same cardiac cycle.
4. *Retained inflow (RI, %)*: percentage of blood that enters the LV during diastole and is not ejected in the following systole.
5. *Residual volume (RV, %)*: percentage of blood that spends more than two cycles in the LV before being ejected.
6. *RV*_*U*_*, RV*_*L*_ *(%)*: residual volume of blood in the upper and lower LV respectively (Fig. 2A).
7. *R*: ratio of DF over RV at peak systole (measure of systolic flow efficiency).

In each patient, all hemodynamic parameters from the predictive models were normalised based on the corresponding value from the baseline model.

### Statistical analysis

Descriptive statistics are presented as mean ± SD. A simple regression model was used to predict the variations of each normalised hemodynamic parameter with OF. Pair-wise comparisons between predictive and baseline models were performed using a Wilcoxon test and differences were considered significant for p < 0.05. A piecewise linear regression of the hemodynamic parameter on OF was also applied to the data to detect the change point (OF_C_, %) at which a change in the slope of the regression line occurred.

### Model validation

The validation dataset (Case 6) included comprehensive imaging data acquired immediately before and after implantation, allowing us to create two independent models pre- and post-TMVR. When in its predictive capacity, the pre-TMVR model included a rigid cylinder with the same internal diameter, frame height and atrial positioning of the implanted valve, a 29-mm Medtronic Intrepid device. The mean and peak PG and obstruction fraction from this predictive model were validated against the values derived from both the post-TMVR imaging data and model (Table 4). A difference of 10%, 14%, and 8% between the imaging data and the predictive model are observed in the end-systolic values of the α angle, C-sept distance, and LV AR, respectively. The same parameters measured in the predictive and post-TMVR validation model showed a difference of 5%, 3%, and 6%, respectively. A comparison of the Doppler-derived data and the predictive model showed differences of 26% and 15% in the mean and maximum PG-LVOT, respectively. Similarly, when the predictive and validation model were compared, the mean and maximum PG-LVOT differed by 3% and 13%, respectively. The maximum flow velocity at the AV plane derived from the Doppler data was 12% lower than the corresponding value in the predictive model and 4% lower than the that from the validation model. Both models showed a maximum OF between 6% and 7%, which was in agreement with the difference in the effective orifice area measured from CTA data pre- and post-TMVR (approximately 9%).

**Table 4:** Model validation.

Parameter	Imaging measurements (CTA, Doppler)	Predictive model (pre-TMVR data)	Validation model (post-TMVR data)
α angle (deg)	142	158	150
C-sept (mm)	3.1	3.6	3.7
LV AR	1.51	1.65	1.55
OF (%)	9	6.9	6.5
Mean PG LVOT (mmHg)	4	2.95	3.04
Max PG LVOT (mmHg)	5.76	6.8	5.94
Max AV velocity (m/s)	2.97	3.38	3.10

α = angle of the aorto-mitral junction at end systole. C-sept = distance between the mitral valve coaptation point and the intraventricular septum at end systole. LV AR = left ventricular aspect ratio at end systole. PG = intraventricular pressure gradients.

## Results

### Impact of TMVR on intraventricular pressure gradients

**Figure 3:**
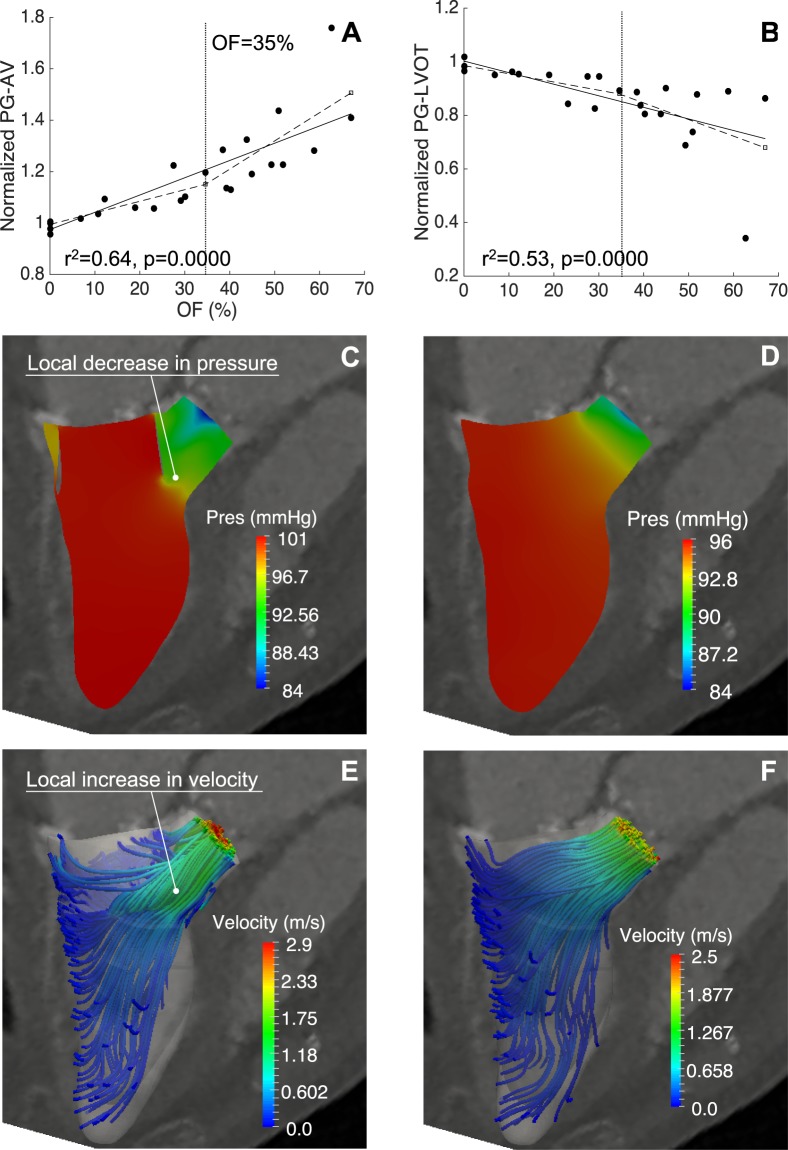
Pressure gradients and flow topology. Apex-to-AV pressure gradients increased from the baseline values after implantation, with a significant change in the growth rate for OF >35% (**A**). The pressure gradients in the LVOT (**B**) decreased with increasing OF, due to a pressure recovery distal to the bottleneck, as typified by the pressure isocontours in Case 1 (**C**,**D**). The blood flow streamlines colored by velocity magnitude illustrate the corresponding variation in flow speed after and before implantation (**E**,**F**).

Simple linear regression was performed to predict the effect of LVOT obstruction on PG-AV and PG-LVOT (Fig. 3A,B).

The normalised peak systolic PG-AV increased monotonically with OF in Fig. 3A (r^2^ = 0.74, p < 0.05) compared to the baseline values (normalised mean 1.18 ± 0.19, p = 0.002). Piecewise regression analysis identified a critical value OF_C_ = 35%, which corresponded to an increase in PG-AV of 21%. Specifically, the mean increase in PG-AV from the baseline was 5.7% for OF <35% and 30.1% for OF >35% (normalised mean 1.06 ± 0.07, p = 0.020 vs. 1.30 ± 0.19, p = 0.028). A polynomial fitting analysis showed that the value OF_C_ = 35% identified a switch from a linear to an exponential growth of PG-AV with OF (r^2^ = 0.91).

Contrary to the trend identified for PG-AV, in Fig. 3B the peak systolic PG-LVOT post-TMVR decreased from the baseline value (r^2^ = 0.53, p < 0.05) over the range of OF tested (normalised mean 0.85 ± 0.15, p = 0.000). A mean decrease of 6.6% for OF <35% and 23.5% for OF >35% was observed (normalised mean 0.93 ± 0.06, p = 0.046 vs. 0.76 ± 0.17, p = 0.036).

Pressure isocontours from the baseline and predictive models for Case 1 typify the disrupting effect of the obstruction on the PG-LVOT (Fig. 3C,D). The corresponding streamlines of blood flow show the velocity increase induced by the artificial narrowing of the LVOT due to the prosthetic valve frame (Fig. 3E,F).

In Case 2 and 6, where no obstruction was observed due to anatomical features, both peak systolic PG-AV and PG-LVOT after implantation were close to the baseline values.

### Impact of TMVR on blood flow components

**Figure 4:**
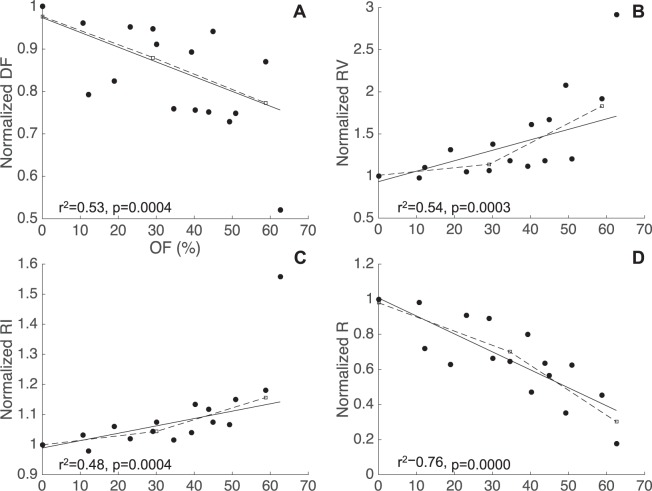
Blood flow components normalised by the baseline values vs. OF. (**A**) Decrease in the DF component (**A**) and a simultaneous increase in the RI and RV components (**B**,**C**) is observed with increasing obstruction, leading to a decrease in the R ratio (**D**). Piecewise regression analysis identified a significant change in trend at OF = 30% for RV and RI, and at OF = 34% for the R ratio. The particle analysis was performed in all patients except Case 6 due to stability issues in the particle-tracking algorithm.

Figure 4A shows that the DF component post-TMVR decreased on average by 13.9% from the baseline value in the range of OF tested (normalised mean 0.86 ± 0.13, p = 0.000). The RV and RI components post-TMVR increased on average by 35.6% and 10.3% respectively from the corresponding baseline values (normalised means 1.36 ± 0.50 and 1.10 ± 0.14, p < 0.005) (Fig. 4B,C).

For all flow components a change point was identified for OF values of approximately 29–30%. Above this OF_C_ the slope of the regression line increased significantly by 46.8% and 37.5% for RV and RI when respectively compared to the corresponding slope of the overall linear regression, while a moderate increase of 3.5% was reported for DF. For OF <30% the mean increase from the baseline in the RV component was 4.4%, but this value raised significantly to 57.5% for OF >30% (normalised mean 1.04 ± 0.14 and 1.57 ± 0.56, p < 0.01). Mean increases from the baseline of 8.9% for OF <30% and of 14.1% for OF >30% were recorded for the RI component (normalised mean 1.09 ± 0.11, p = 0.0124 vs. 1.14 ± 0.15, p = 0.0009).

Overall the R ratio in the post-TMVR models decreased from the baseline by 36.7% with increasing OF (normalised mean 0.63 ± 0.22, p < 0.0001) (Fig. 4D). Piecewise regression identified an OF_C_ = 34%, which corresponded to a decrease of 31%. The mean decrease was 17.4% for OF <34% and 46.2% for OF >34% (normalised mean 0.83 ± 0.15 vs. 0.54 ± 0.18, p < 0.0001).

**Figure 5:**
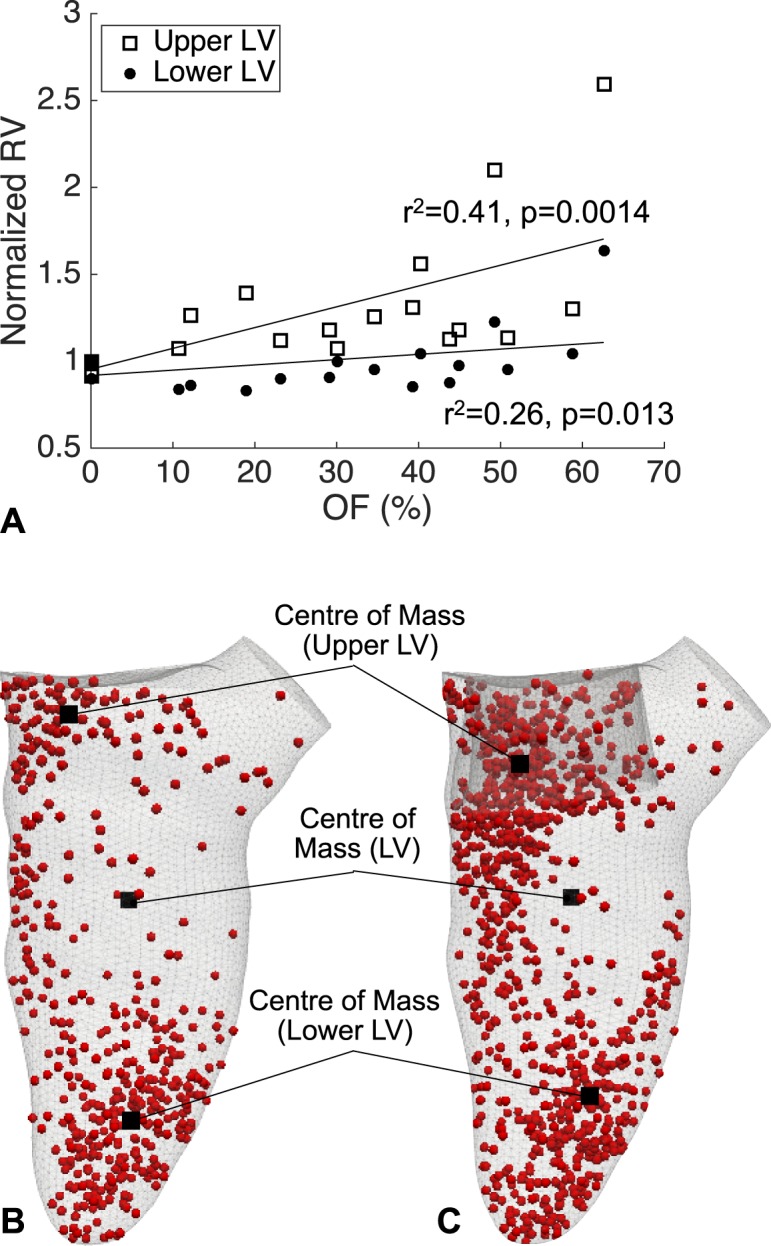
Residual particle distribution in the LV. (**A**) The RV component increases with obstruction in the upper LV, while no significant difference is observed in the lower LV after implantation. (**B**,**C**) A snapshot in late diastole in Case 1 in the baseline and predictive model (OF = 49.3%) shows a clear accumulation site near the implanted valve in the upper LV.

The post-TMVR RV_U_ component increased on averaged by 27.1% from the baseline in the range of obstructions tested (normalised mean 1.27 ± 0.43, p = 0.0014), while RV_L_ component did not show any statistically significant variation (Fig. 5A). A comparison of the RV particle distribution in the baseline and predictive model for Case 1 showed a marked accumulation of RV particles between the septal wall and the valve frame and inside the valve frame in the predictive model, while a similar distribution was observed in the lower LV of both models (Fig. 5B,C).

## Discussion

Our results show that an LVOT obstruction greater than 35% is associated with deleterious effects on ventricular hemodynamics following TMVR, including a significant increase in ventricular afterload and in the blood volume residing inside the ventricle for more than two cycles.

Previous studies on LVOT obstruction found that a 24% reduction in the AV indexed effective orifice area (EOA) resulted in a 33% increase in transaortic pressure gradients, which increased exponentially with EOA decreasing below a threshold value^25,26^. An acute afterload increase of 30% was also associated with sudden death and profound functional and anatomical changes in 7 closed-chest porcine models^27^. Our results consistently show an exponential increase in afterload for LVOT area reductions greater than 35%, with a mean increment in the peak systolic pressure gradient of 30.1% in the upper range of obstruction tested (Fig. 3A). Since our predictive and baseline models are both based on the same wall motion (i.e. volume change) and systolic duration, in the presence of LVOT obstruction the systolic pressure gradient must increase to achieve the same cardiac output in the same length of time. If persistent, this compensatory mechanism can introduce adverse ventricular remodelling leading to progressive heart failure. In particular, increases in systolic pressure gradients and afterload can trigger adverse hypertrophic remodelling. In turn, a hypertrophic septum bulging into the ventricular cavity can exacerbate LVOT obstruction and further increase the afterload, causing a negative feedback mechanism. However follow-up studies are needed to identify the level of LVOT obstruction that promotes transition from an adaptive to a maladaptive ventricular remodeling.

Outflow obstruction also resulted in a localised decrease in the systolic pressure gradient in the LVOT that was not present in the baseline models, where the pressure changes occurred gradually in space (Fig. 3D). The predictive models showed that the narrowing due to the valve frame caused a local increase in the blood flow velocity, thereby generating a sharp pressure decrease in the bottleneck region as postulated by the Bernoulli principle (Fig. 3C,E). The magnitude of this pressure recovery depends on the amount of energy dissipated in the blood as it flows through the bottleneck, which in turn is affected by the neo-LVOT shape and the blood velocity. This spatial variation of the pressure gradient can represent a confounding factor in the LVOT assessment using Doppler measurements^28^.

The afterload increase necessary to maintain the cardiac output also affects the blood residence time in the LV: the degree of LVOT obstruction that triggered hemodynamic deterioration was consistent across blood residence times and pressure gradients estimates at 30–34% and 35% respectively. The blood residence time increased for all models following implant due to a shift from cardiac output being predominantly driven by the direct flow component to increasingly being a result of retained inflow and residual volume from earlier cycles being ejected. A significant increase of 57.5% in the RV component was observed in the higher OF range, indicating a deterioration of systolic flow efficiency that is proportional to the degree of LVOT obstruction. This finding is in agreement with results from previous *in-vitro* models, which showed blood accumulation sites near the valve structure and between the valve frame and the myocardium, creating potential sites for thrombus formation^29^. Such a conclusion is also supported by our finding of an increase in the number of RV_U_ particles post-TMVR, which was not accompanied by a consistent decrease in the number of RV_L_ particles. In the predictive models, these extra RV_U_ particles accumulate in the sites between the myocardial wall and the valve or inside the valve frame itself (Fig. 5C), while no significant difference is observed between the baseline and TMVR groups in the accumulation site of the lower LV, i.e. the apical region.

The goal of this study was to quantify the level of LVOT obstruction that is likely to impair ventricular hemodynamics. In this context, the heterogeneity of our cohort in terms of baseline characteristics and type of implanted device does not subtract from our main finding. Simulation results are only dependent on the aspect ratio of the device (H/D) and do not account for specific manufacturing characteristics. This approximation allows the models to be consistent across implants and thus does not limit the generalisability of the results. The simplified valve represents the bulk of frame and leaflets without distinguishing between each structure: while such representation is an approximation of the real system, this is what eventually causes obstruction in the LVOT, whose prediction is the main goal of the study. However, further modelling work on a larger number of patients will be necessary to investigate how anatomical parameters such as the size of the LV cavity may affect the threshold OF for haemodynamics deterioration, since LVOT obstruction can impact the behaviour of the LV differently in small ventricular cavities. Similarly, future modelling studies with more realistic shapes of implanted device should also be performed to analyse the effect of the bioprosthesis geometry on ventricular haemodynamics. This will be highly relevant to the topic of LVOT obstruction, as new dedicated D-shaped MV bioprostheses that are able to mimic the original shape of the mitral annulus, and thus avoid high transvalvular gradients by maximising the inflow area, are also likely to protrude into the LVOT causing obstruction due to their large size. While smaller devices like the AV bioprostheses deployed in the mitral position may circumvent this risk, they are also prone to stability issues and patient-prosthesis mismatch, underscoring the need for robust means to match device model and size to the individual patient’s pathophysiology.

Finally, the ventricular geometry in our models did not include trabeculations, chordae and papillary muscles and thus the respective effects on the blood flow could not be investigated. However, the tracked wall motion from the CTA data incorporates the effects of these structures on the global and segmental wall motion, as well as those due to wall thickness, potential prior myocardial infarction or any other contraction defect, which would also result in an alteration of the motion pattern derived from the imaging data. Similarly, as direct *in-vivo* measurement of pressure were not available, we used experimentally measured velocity profiles at the valve planes that account for both preload, afterload, and potential altered blood flow dynamics such as aortic stenosis or concomitant mixed valve disease. While the present study only provides a proof of concept of the potential of this modelling approach, the impact of contraction patterns, ventricular loading, and geometries of the subvalvular apparatus on LV function post-TMVR can be investigated in further work using this methodology by modelling a cohort of patients of sufficient sample size to represent significant variations in all these factors.

## Conclusion

By combining imaging and computer modeling we have provided a tool to predict LVOT obstruction post-TMVR and to quantify the impact of varying degrees of obstruction on ventricular afterload and blood residence times, providing a link between anatomical and hemodynamics parameters. This approach overcomes some of the limitations of current methods for LVOT obstruction predictions, which largely rely on anecdotal experience and standard anatomical evaluation. The goal of our modelling technique is to create impact on patient care and procedural skills by informing the device choice and placement to lower the possibility of reinterventions. This is particularly relevant in TMVR, where reintervention is associated with high morbidity and mortality rates. Despite the small size and heterogeneity of the cohort analysed, these results show a clear correlation between the degree of LVOT obstruction and changes in ventricular afterload and blood residence times following TMVR. A LVOT obstruction between 30% and 35% has been identified as a threshold for hemodynamic deterioration, providing a quantitative criterion for intervention that has been lacking so far. The next step for an effective clinical translation of this technology will be to apply it to a large number of patients to generate detailed response maps, which could be used in the future to design and test new devices.

## Data Availability

According to UK research councils’ Common Principles on Data Policy, all data supporting this study will be openly available at 10.18742/RDM01-408.
